# Ultrafast laser processing of materials: from science to industry

**DOI:** 10.1038/lsa.2016.133

**Published:** 2016-08-12

**Authors:** Mangirdas Malinauskas, Albertas Žukauskas, Satoshi Hasegawa, Yoshio Hayasaki, Vygantas Mizeikis, Ričardas Buividas, Saulius Juodkazis

**Affiliations:** 1 Laser Research Centre, Department of Quantum Electronics, Physics Faculty, Vilnius University, Saulėtekio Ave. 10, LT-10223 Vilnius, Lithuania; 2 Center for Optical Research and Education (CORE), Utsunomiya University, 7-1-2 Yoto, Utsunomiya 321-8585, Japan; 3 Research Institute of Electronics, Shizuoka University, 3-5-3-1 Johoku, Naka-ku, Hamamatsu 432-8561, Japan; 4 Centre for Micro-Photonics, Faculty of Science, Engineering and Technology, Swinburne University of Technology, Hawthorn, VIC 3122, Australia; 5 Melbourne Centre for Nanofabrication, ANFF, 151 Wellington Road, Clayton, VIC 3168, Australia; 6 Center of Nanotechnology, King Abdulaziz University, Jeddah 21589, Saudi Arabia

**Keywords:** biomedical applications, direct laser writing, functional microdevices, material processing, nonlinear light–matter interaction, 3D structuring, ultrashort laser pulses

## Abstract

Processing of materials by ultrashort laser pulses has evolved significantly over the last decade and is starting to reveal its scientific, technological and industrial potential. In ultrafast laser manufacturing, optical energy of tightly focused femtosecond or picosecond laser pulses can be delivered to precisely defined positions in the bulk of materials via two-/multi-photon excitation on a timescale much faster than thermal energy exchange between photoexcited electrons and lattice ions. Control of photo-ionization and thermal processes with the highest precision, inducing local photomodification in sub-100-nm-sized regions has been achieved. State-of-the-art ultrashort laser processing techniques exploit high 0.1–1 μm spatial resolution and almost unrestricted three-dimensional structuring capability. Adjustable pulse duration, spatiotemporal chirp, phase front tilt and polarization allow control of photomodification via uniquely wide parameter space. Mature opto-electrical/mechanical technologies have enabled laser processing speeds approaching meters-per-second, leading to a fast lab-to-fab transfer. The key aspects and latest achievements are reviewed with an emphasis on the fundamental relation between spatial resolution and total fabrication throughput. Emerging biomedical applications implementing micrometer feature precision over centimeter-scale scaffolds and photonic wire bonding in telecommunications are highlighted.

## Uniqueness of Ultrafast Laser Processing

The possibility of three-dimensional (3D) writing in glass^1^ and polymers^2^ using tightly focused femtosecond (fs) laser pulses, demonstrated roughly two decades ago, has attracted attention in a wide range of areas related to academic research and engineering. The fabrication of 3D objects with a size comparable to that of a living cell and comprising even finer details^3^ suggests a realization of remotely controllable 3D micro-bots to perform *in vivo* healing missions or the creation of all-optical information processors integrated on a single 3D microchip and robust non-erasable optical memory structures. Thus began the race towards these and many other attractive goals. At present, most of those goals have yet to be reached; however, the progress achieved in some areas is significant. Currently, optical memories with data density exceeding ∼1 Tbit cm^–3^ (Refs. 4, 5), waveguide-based optical information processing structures^1^, elements of optical quantum computing systems^6, 7^, 3D photonic crystals (PhC)^8^ and micro-mechanical/biological systems^9, 10^ are obtainable using ultrashort laser pulses. Here, we discuss these achievements and outline current trends in the development of laser processing and its applications, which are bound to make ultrashort laser fabrication an indispensable tool for future nanotechnologies.

Unrestricted freeform manufacturing in 3D-space on the mesoscale spanning the critical dimensions of 10 nm to 100 μm in feature sizes has been an engineering curiosity over the last 10–15 years. Endless incarnations of micro-copies from real-world items were fabricated in a variety of photo-polymers, recorded in glasses and crystals. This endeavor pushed and explored the limits of novel 3D fabrication and experimented with optimization for a higher throughput and resolution^11^. The efforts continue to this day and challenge well-established benchmarks in resolution, feature size, precision and efficiency developed over more than 50 years in the microelectronics industry. Comparison of 3D emerging technology with a mature two-dimensional (2D) micro-technology is only partially fair owing to a qualitative difference in the 3D character and the capability of laser manufacturing. Both approaches have fundamental limitations set by the wavelength of fabrication for electrons and photons, respectively. The difference in the relevant wavelengths is revealing: the electron’s de Broglie wavelength at a typical *V*=50 kV acceleration voltage in electron beam lithography is 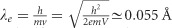 (nonrelativistic), where *h*, *e*, *m* are the Plank’s constant, electron charge and mass, respectively, whereas the light wavelength *λ*_l_=5000 Å (a green color) in 3D-laser writing.

Lasers entered and now dominate the fields of welding, drilling, cladding and manufacturing with a unique capability of 3D robotic light delivery at a 0.1–10 m scale and pointing stability of ∼1 mm (for example, in the car manufacturing industry) since circa 1980 (Ref. 12). Lasers operating at 1 or 10 μm wavelengths and at long pulse or continuous-wave (cw) mode are practical and useful, yet unsuitable for fabrication tasks at scales smaller than 1 mm. Thus, they are presently challenged in precision and resolution by reliable ultrashort pulsed lasers. A double innovation in (i) the development of new materials and (ii) laser sources that are better suited for 3D micro-printing was required for the continued progress of laser fabrication and the long-praised resolution of fabrication not being the driving factor.

Now, with the emergence of a new generation of reliable fs-lasers, a set of new materials and backing by science of light–matter interaction at the nanoscale with cross-sections smaller than 100 nm, miniaturization of 3D-laser fabrication is advancing and innovating. To demonstrate the versatility and potential of the new technology, 3D inscription, additive manufacturing and surface texturation techniques are shown to reach the high throughput required for industry. A selected few representative examples illustrate the capabilities and truly unique features of fs-laser fabrication.

## Is Fabrication Using Ultrafast Lasers A Future Technology?

### Industrial challenges

Manufacturing has its own conservative pace of development and justification of new investments. Considerable innovation that brings a new quality to the product simultaneously with higher productivity is always required for the next cycle of technological renovation, which occurs on a 7–10-year-modernization cycle on a factory floor. In manufacturing with lasers, their acquisition and maintenance costs, reliability and longevity were always the main driving forces for industrial installations. The innovation that an ultrashort pulsed laser can bring to manufacturing is discussed next from the perspective of unique control over the light–matter interaction when ultrashort laser pulses are used and in view of the practical requirements of high productivity.

**Figure 1:**
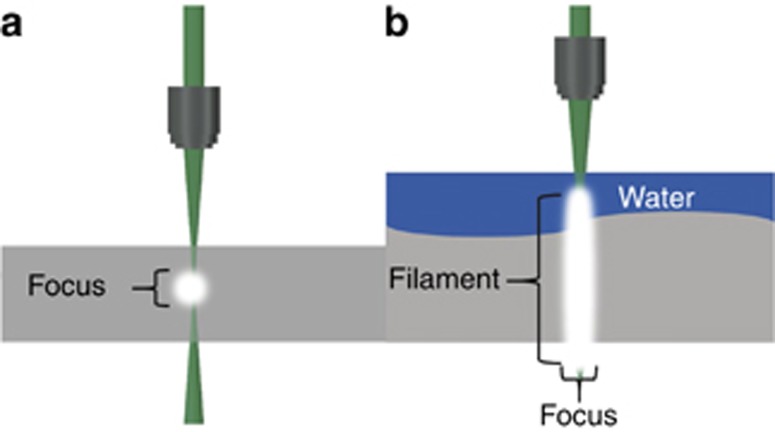
Beam focusing approaches: (**a**) a beam is focused on the surface or volume of a material; (**b**) an fs-beam is focused on liquid and forms a light filament suitable for processing of transparent and opaque workpieces at different axial locations or curvature of the surface. Reproduced with permission from Ref. 13. JLPS. All rights reserved.

The additive manufacturing, cutting and welding with CO_2_, YAG and fiber lasers that operate at 10 or 1 μm wavelengths with a cw or pulsed-mode operation are already powering the automotive, construction and marking industries^12^. The feed speeds of a workpiece are ∼10 cm min^–1^ (∼1 mm s^–1^ or in specific cases even higher) for marking and welding applications realized with lasers with an average power of ∼0.1–1 kW. The target industrial macro-fabrication speed is ∼10 cm min^–1^ for a linear scan, including turning points, which can already be demonstrated in fs-laser micro-fabrication^13, 14^ with a recent record in waveguide writing speed of 200 mm s^–1^ (Ref. 15) on the screen of a mobile phone inside Gorilla glass. Another efficient and confined light delivery is a filament^16^ self-formed in water, which can assist in uniform energy delivery along the beam propagation direction^17^. This helps laser fabrication of curved surfaces where tight focusing would require axial position adjustments for precise matching of the focal region and the sample’s surface^18^. The principle of this approach is depicted in Figure 1. The formed filament can have a high aspect ratio (length to width) and experimentally realized more than 2 mm in length and just tens of micrometers in diameter^13^. When a laser raster scan or multi-line exposure mode is used for a fill exposure of 3D volume of a workpiece/pattern, the fabrication time quickly increases to hours for an object with 3D cross-sections of ∼1 in.

The fastest available beam scan approach employs mechanical galvo-scanners that can deflect the beam even faster than 10 m s^–1^. However, such high-speed scanning is achievable only for linear structures. When turning tight corners, acceleration becomes a serious issue. The acousto-optical device-based deflector enables increased beam scanning velocities up to 2 m* *s^–1^ with no inherent limitations on a turn radius maintaining high positioning repeatability^19^. In addition, powerful laser sources can be used for parallel multibeam^20^ fabrication as well as employing passive^21^ or active beam shaping techniques^22^. Such implementations dramatically increase fabrication throughput. Recently, tomographic data were used as a master CAD model for laser 3D fabrication^23^, an approach adopted from 3D printing.

Fs-laser fabrication have application where high precision is required, where structuring of the surface and bulk of transparent materials that are brittle and hard must be performed. If composite and layered materials must be structured in a complicated 3D fashion, it can be performed by fs-laser structuring. This has been understood from the basics of light–matter interaction in which pulses shorter than the energy relaxation between electrons and lattice ions can deliver energy with the highest precision and without plasma screening/reflection effects^24, 25, 26^.

### Scaling from 2D to 3D

Because high precision and small feature size are incompatible with large-throughput fabrication—Tennant’s law^27^—and by following the universal trend of miniaturization, we expect future applications for fs-laser manufactured structures and objects to be used in smaller-footprint lab-on-a-chip applications. Lab-on-a-chip devices have typical dimensions of 1 × 1 cm^2^ and feature sizes of the functional elements such as channels and optical elements on the order of a cross-section of an optical fiber (a human hair) of ∼100 μm. At this already challenging scale, the surface finishing and smallest structures should be controlled at *λ*/20 nm precision/resolution for optical functionality.

Fs-laser sources at 50–1000 kHz repetition rates and average power up to 10 W for a typical wavelength *λ*≃1 μm and pulse duration *τ*_*p*_≃200 fs have reached an industrial grade in terms of reliability and capability of fabrication at the required ∼10 cm min^–1^ workpiece feed rates. Tennant’s law linking the resolution, *R* (nm) and the throughput, *T*_2D_ (μm^2^ h^–1^) of fabrication via a planar lithography predicts the following scaling^27^:


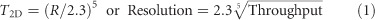


**Figure 2:**
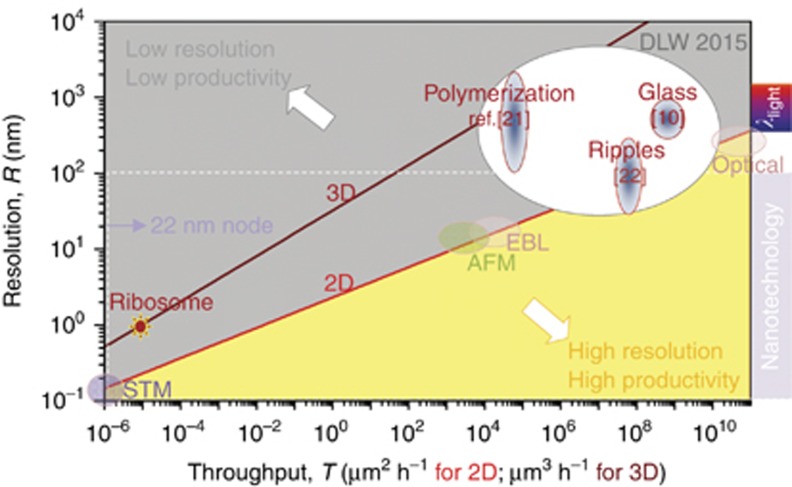
Tennant’s scaling: state-of-the-art of nanotechnology in 2003 for the resolution vs. throughput *R*=2.3^5^ *T*_2D_ (Ref. 27). Direct 3D laser writing for polymerization^28^ with *T* ≃ 10^9^ μm^3^ h^–1^, Bragg grating recording by Bessel–Gauss fs-beam in glass^14^, surface ripple formation on crystals/glasses^29^ are marked along with the Tennant’s law predictions rescaled for the 3D *C* × 10^6^ μm^3^ h^–1^ with *C*=32.2 taken from the speed of protein production in ribosome^30^ (see text for details). The size of elliptical markers is representative of the resolution span; the current 22 nm node of modern CMOS lithography is marked by an arrow.

Following the arguments of scaling, for a 3D fabrication via lithographic approach, one could expect 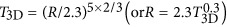 with *T*_3D_ (μm^3^ h^–1^), which follows from area-to-volume scaling. However, Tennant’s law is for the 2D fabrication; for the production of 3D objects, we use a more general expression 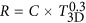, where value of *C*=32.2 is taken to pin the power plot (Figure 2) to the natural throughput of protein production in a 3D conformation by a ribosome; the resolution (often referred to but not to be mixed up with the individual feature size) is taken as 1 nm. This *C* value corresponds to the production rate of 20 amino acids per second in a cell^30^. It is noteworthy that this comparison of direct writing used in fabrication—for example, building 3D objects by laser polymerization^28^ or glass structuring^29^—is valid because ribosome is also constructing 3D proteins from a linear code^30^. Another measure of the 3D production rate can be made from the growth rate of cells. Human nails grow at a 1 nm s^–1^ linear speed, which means 3.6 μm^3^ h^–1^, and is similar to the protein production rate.

Figure 2 shows typical Resolution ∝ Throughput regions marked by ovals for the manipulation of single atoms by scanning tunneling microscopy (STM), writing by oxidation of SiO_2_ with an atomic force microscope (AFM) needle, standard electron beam (EBL) and optical mask projection lithographies. These are 2D technologies that obey Tennant’s scaling *T*_2D_=(*R*/2.3)^5^, which is a pure empirical dependence. Interestingly, starting from the current 22-nm node of modern complementary metal oxide semiconductor (CMOS) lithography, the field transistor design allows the exertion of 3D control of the depletion regions in the channel. Hence, the future of planar 2D technology tends to become 3D functional as the feature size is reduced^31^. Apparently, for the 3D case (Figure 2), direct laser writing by polymerization^28^ and volume Bragg gratings formed by axially extended Bessel–Gauss fs-beam^14^ delivers a 3D finished and functional structure. For example, direct laser writing with fs-laser pulses delivers 3D fabrication of ∼10^3^ μm^3^ s^–1^ with focal spot size *d*=1.22*λ*/NA≃*λ* for 3D structuring of silicone elastomer^28, 32^; here, NA is the numerical aperture of the objective lens. On the Resolution ∝ Throughput plot (Figure 2), the corresponding 3D fabrication examples tend to cluster towards the desirable lower-right side of the lines representing Tennant’s law for the 3D case. This shows that for 3D-laser fabrication, the throughput can meet industrial standard at the required resolution for 3D manufacturing.

## Ultrafast Laser Lithography

The trend of miniaturization, which began in CMOS electronics, is currently spreading to other areas, such as micro-optics, micro-fluidics and micro-mechanics. In these fields, new functionalities of micro- and nanostructures stem from physical or chemical responses arising because of size and shape-related effects and, in the case of optical systems, benefit from enhanced interactions between light and matter in artificially structured media. Perhaps the most intuitively appealing examples of such applications are optically controlled mechanical phenomena—for example, laser trapping—which rely on opto-mechanical susceptibility of microscopic particles and structures to optically generated minute forces and torques. Other rapidly evolving areas are optics of plasmonic nanoparticles and PhCs, which enable control over light emission, absorption and propagation at the nanoscale, a demand strongly stimulated by the need for green and sustainable use of energy and materials. Below, we will mainly focus on the structuring of dielectrics and their new optical, mechanical and biomedical functionalities.

### Requirements for surface quality

Micro- and nanofabrication of optical structures requires surface roughness <*λ*/20, (≃20−40 nm for visible wavelengths) to reduce random light scattering. Although this requirement can be in some cases alleviated by post-fabrication treatment—for example, controlled remelting, lapping and polishing of microscale objects—it still remains a difficult task. Therefore, the fabrication techniques capable of directly delivering the required quality are essentially preferred. In this respect, the potential of fs-laser lithography has been demonstrated many times, and some examples will be given in the sections below. In general, the surface quality of laser-fabricated optical elements is high enough to enable their direct use in photonic applications.

### Materials and processing techniques

Owing to the requirements of high surface quality and low random scattering, laser fabrication of optical micro- and nanostructures prefers nondestructive photomodification processes over optical breakdown or other destructive processes. Therefore, liquid photocurable organic resins and negative-tone photoresists are mainly used as initial materials for direct laser writing (DLW) lithography. In these materials, optical absorption induces photochemical reactions, such as photopolymerization (curing). In liquid resins, this process leads to liquid-to-solid transition, whereas in photoresists—which in most cases are exposed as dry, solidified films—photomodification has a latent character. After the laser structuring, unexposed liquid resin is rinsed away, whereas unexposed resist is dissolved in a developer and removed from the structure. Finally, the rinse liquid is dried; this is a critical step for finely patterned, fragile structures, because they may become completely destroyed by powerful capillary forces. These undesired effects can be suppressed by the use of freeze-drying or critical-point drying techniques to eliminate the surface tension effects^33, 34^.

Historically, liquid resins were the first materials to show potential for 3D-laser structuring^2, 35, 36, 37^, but later negative-tone photoresists became widely used owing to their higher stability during processing and lower post-drying shrinkage. Among photoresists, epoxy-based chemically amplified photoresist SU-8 has acquired wide popularity^8, 38, 39, 40^, because this resist, specifically intended for microfabrication of mechanical components via ultraviolet lithography, was already commercially available during the early steps of development of 3D-laser lithography. SU-8 is optically transparent in visible and infrared (IR) spectral ranges, but can be exposed via two-photon and multi-photon absorption, ensures submicron resolution, has a refractive index ≈1.5, and is mechanically strong and biocompatible. Numerous demonstrations of micro- and nano-photonic structures—for example, 3D PhCs—fabricated in SU-8 can be found in the literature^41, 42^.

Subsequently, new classes of photoresists specific for laser lithography were developed, aiming to further improve spatial resolution, simplify post-processing and reduce drying-related shrinkage. A class of photoresists based on a zirconium propoxide sol–gel^43^ developed by a collaboration of several groups offers significantly improved spatial resolution, low proximity effect (thin threads that evolve randomly and uncontrollably between closely spaced larger features) and low shrinkage^44^. This is achieved by combining organic and inorganic components and various modifications of this hybrid material, also known as SZ2080, which were synthesized to target specific purposes—for example, assisting subsequent metallization by the inclusion of metal-binding moieties^45^ and the improvement of spatial resolution by inclusion of mobile quenching molecules^46^. Tuning proportions of photoresist components, such as glass-forming silica, zirconia, titania and methylmetacrylate plastic-like moieties allow a flexible choice of mechanical and optical properties of micro-optical elements^47, 48^. In addition to SZ2080, a line of IP resists developed by and available commercially from Nanoscribe GmbH^49^ offers similar advantages for ultrafast laser structuring. IP photoresists also include liquid photoresists, which can simultaneously act as an index-matching liquid during the DLW process and has enabled the achievement of structures with extremely large heights^50^. Because swelling and shrinkage during development and drying is inherent to negative-tone photoresists^33, 51, 52, 53^, shrinkage effects can be significantly suppressed by using positive-tone resists borrowed from the semiconductor industry^54, 55^; however, in this case, the total exposed (to be removed) volume may increase, and the fabrication time may lengthen somewhat in the case of sequential DLW processes. Photosensitivity of photoresists and resins was conventionally tailored by adding photoinitiators to these materials. Recently, careful control over spectral and temporal characteristics of fs-laser pulses has enabled precise control over photo-excitation via multi-photon and avalanche ionization as well as by controlling the thermal conditions at the focal volume inside a pure resist. A combination of high pre-breakdown irradiance and fast scanning opens the possibility to tame chemical-free radical creation and their thermal cross-linking without the necessity of a photoinitiator^11, 56^. Coincidentally, initiator-free cross-linking in silicone^28^ has produced biocompatible material free of the bio-toxic cyclic compounds present in most photoinitiators. Strongly 3D-localized thermal curing of resists^57, 58^ can now be widely explored for microfabrication owing to their fast scan capabilities.

With regard to fabrication techniques, the two main methods are holographic fabrication^20, 21, 22^ and DLW^59, 60, 61, 62, 63, 64^. Holographic fabrication exploits nondestructive exposure of a material to a periodic interference field of several coherent laser pulses, and can be implemented using a simple opto-mechanical setup and a wide variety of amplified and unamplified laser sources. Various implementations of DLW are described in the available literature^11, 65, 66, 67^, and DLW setups are commercially available from several companies^49, 68, 69, 70^. Among the current developments in the practical implementation of the DLW technique is a significant increase of the laser beam scan speed in the sample from ∼100 μm s^–1^ toward meters-per-second achieved via the combined use of fast mechanics for sample translation and scanning of the beam focus inside the sample^9, 71, 72^. Special exposure schemes tailored to improve the axial resolution of DLW can also be added^73, 74, 75^. For exposure of photoresist or resin samples, fs-laser oscillators (for example, Ti:sapphire or fiber laser systems) provide sufficient exposure levels. The wide availability and constantly decreasing size and price of these laser sources contribute to the practical applicability of the DLW technique. For exploitation of the DLW technique near or above the laser-induced breakdown threshold in solid crystals or glasses (for example, for waveguide writing and creation of new materials), amplified laser systems providing pulse energies >1 μJ are required.

**Figure 3:**
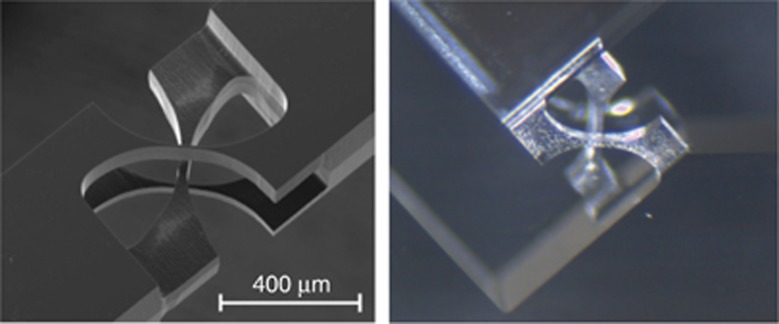
A cross-pivot hinge out of fused silica as a part of a mechanical structure. The images show the three crossed beams and the rounded corners at the location where the beams are connected to the main body. Note the high aspect ratio of the micromachining process. Reproduced with permission from Ref. 76. MDPI. All rights reserved.

Next to lithography, the fs-laser irradiation can be very similarly applied for processing of fused silica (the amorphous form of SiO_2_) and introduces a novel technology platform for highly integrated all-optical microsystems. In contrast with common approaches that rely on combining materials to achieve particular functions, fs-laser-fabricated microsystems rely on single material monolith, whose properties are locally and 3D functionalized by selective exposure. The combination of functionalized zones with different physical properties allows integration of systems without the need for further assembly of packaging steps or without the need for multiple processing steps, like for instance sequences of layers deposition, exposure and etching steps (Figure 3; Ref. 76). Lastly, there is numerous other additive (and subtractive) manufacturing approaches where ultrashort pulses have significant advantages over longer pulses. To mention a few techniques like laser-induced forward transfer (LIFT) or an intense field-induced self assembly are not reviewed here; see Refs. 77, 78 for the detailed account.

### 3D micro-optical elements

**Figure 4:**
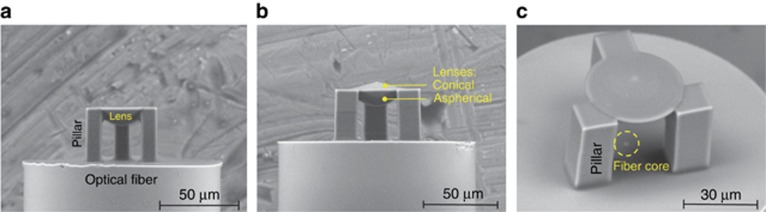
Freeform 3D micro-optical elements fabricated by DLW: hybrid optical elements—aspheric and axicon lenses on a tip of optical fiber. Reproduced with permission from Ref. 81. JLPS. All rights reserved.

**Figure 5:**
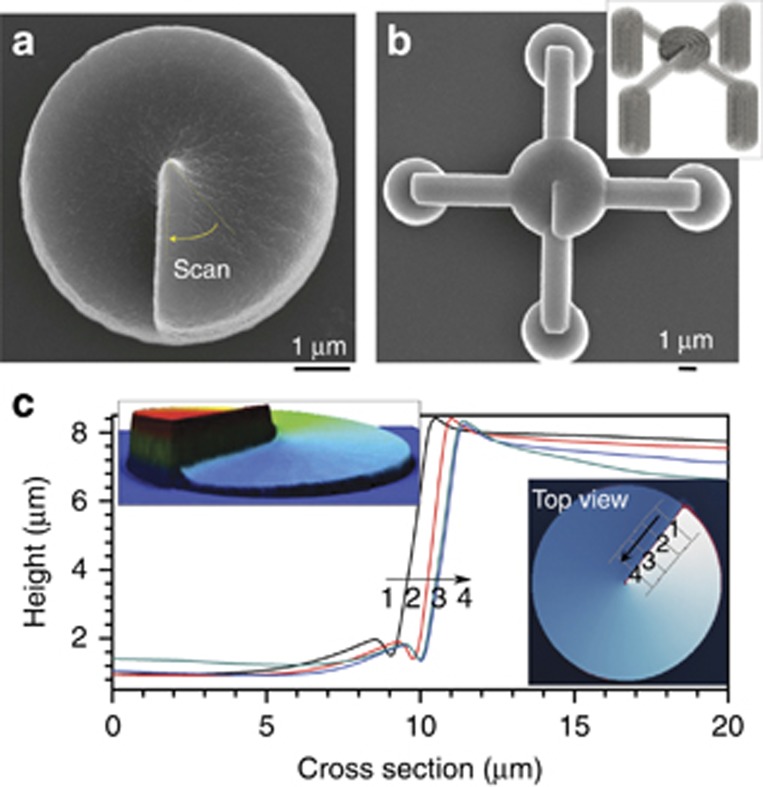
Optical vortex generating spiral waveplates. Smaller (**a**, **b**) and larger (**c**) cross-sections optical vortex generators. (**a**, **b**) SEM images of micro-plates made by point-by-point exposure (inset in **b** shows 3D construction of irradiation matrix). (**c**) Height scans at four locations of the step edge of the spiral plate; diameter of the plate was 60 μm. Reproduced with permission from Ref. 59. Copyright (2010), AIP Publishing LLC.

Aspherical and axicon micro-lenses as well as diffractive optical elements were DLW fabricated directly on the core of the optical fiber^79, 80, 81^, illustrating the potential of the technique to integrate various components. Figure 4 shows an example of such optical elements fabricated in photoresist SZ2080. Spiral waveplates^59, 82^ for optical vortex generation (Figure 5) and hybrid refractive-diffractive elements blending diffraction gratings and lenses can be made^83^. A rapidly growing field of singular optics exploring the generation and control of optical vortices at a microscale benefits from DLW via polymerization owing to a simple method to achieve ∼100% efficiency of the optical vortex generation. Alternatively, planar patterns for the vortex generation—*q*-plates—are made of azimuthally patterned form birefringence inside glass by fs-laser structuring^84, 85^; however, they suffer from scattering losses.

It is helpful to stress here that these structures clearly satisfy the high surface quality requirements outlined in the section above and are directly usable. It has been already proved by several studies that there is no shape limitation or restriction of a single element in an array of micro-lenses^86, 87, 88^. Obviously, surface quality and optical characteristics of these elements can be retained during their replication—for example by nanoimprint, hot embossing and molding—directly or via an Ni-shim replication^89, 90^. Recently, integration of 2D and 3D optical components comprising both passive and active functionalities—namely, a rectangular waveguide of rectangular cross-section that can be *π*/2-twisted to form an arch on the substrate—was demonstrated^91^. Thus, the application of ultrashort laser processing can lead to realization of new optical integrated devices with novel functionalities such as optofluidic single-cell counters^10^. Interestingly, by applying different exposure doses, one can achieve 3D or 2D varying material properties, thus producing monolithic components with composite properties^92^. This seems to be an attractive option for producing custom-made freeform gradient refractive index micro-optical elements^93^.

**Figure 6:**
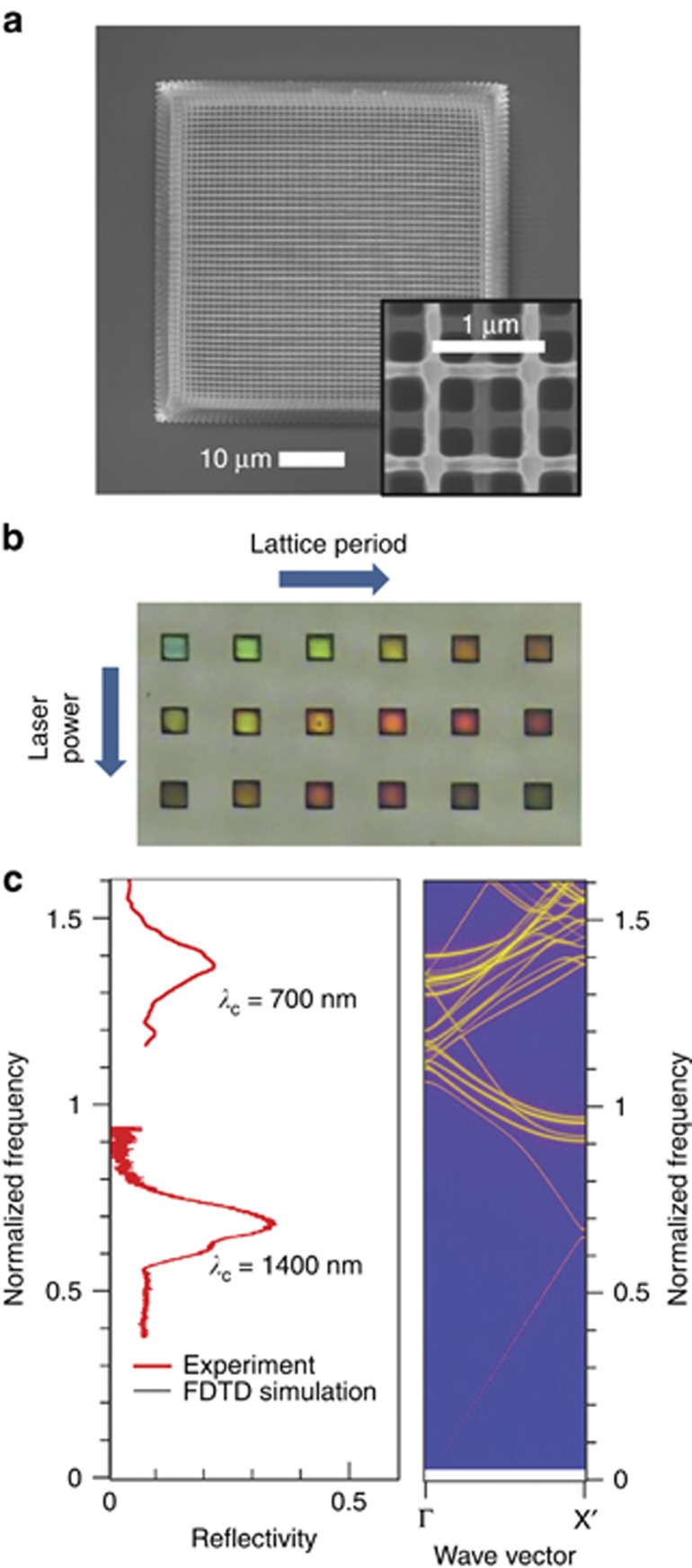
Coloration via slowing light. (**a**) SEM image of 3D woodpile architecture PhC in photoresist SZ2080, (**b**) optical microscopy image of several woodpile structures having different lattice parameters and exhibiting different structural color, (**c**) comparison between experimental optical reflectivity spectrum of the sample shown in **a** and numerically simulated reflectivity spectrum as well as photonic band diagram. Reproduced with permission from Ref. 94. JLPS. All rights reserved.

One of the primary application areas of ultrafast laser lithography in photoresists and resins has been the fabrication of PhC structures^94, 95, 96, 97, 98, 99, 100, 101^, especially 3D ones that are difficult or impossible to realize by other available techniques. 3D PhCs with woodpile^41, 102^, spiral^103, 104^, slanted-pore^105^ and other architectures^64, 106^ with high optical quality were demonstrated during the last decade. Figure 6 summarizes the structural and optical properties of a 3D woodpile architecture PhC fabricated in SZ2080 photoresist by DLW and dried in supercritical CO_2_. Scanning electron microscopy (SEM) images shown in Figure 6(a) demonstrate the DLW fabrication of highly resolved features, evident from woodpile rods with lateral thickness of ~*a*_*xy*_=135 nm. The in-plane lattice period (distance between two neighboring woodpile rods), designed to be 1000 nm, is in reality reduced to 940 nm owing to shrinkage of ~ 5% of the PhC lattice. Shrinkage by a similar amount is also evident in the large-scale SEM image in Figure 6(a). The distance between the neighboring woodpile planes is designed to be 
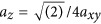
 to maintain a face-centered cubic (fcc) lattice symmetry.

PhC structures with similar parameters were unexpectedly found to exhibit strong resonant reflection at visible wavelengths, although neither fundamental nor higher-order photonic stopgaps (PSG) are expected to open in this spectral range owing to a fairly large PhC lattice period. Figure 6(b) shows optical image of several fcc woodpile structures similar to that in Figure 6(a), having lattice periods *a*_*xy*_ varying in small steps and fabricated at different average powers of the writing laser beam. Observation in reflection mode within an angular incidence range of ±17° clearly reveals the structural color of the PhC structures. The color exhibits redshift with increasing lattice period and writing laser power (that is, dielectric filling ratio) in qualitative agreement with Maxwell’s scaling behavior^107^, thus indicating that its origin is linked to the periodicity of the PhC. Figure 6(c) summarizes the interpretation of the structural color by comparing the experimental reflectivity spectrum of the PhC structure in Figure 6(a) with the reflectivity spectrum and photonic band diagram simulated using the finite-difference time-domain technique. As can be seen, a fundamental PSG opens along the observation (woodpile layer stacking) direction near the 1400 nm wavelength in the only PSG and results in a near-IR reflectivity band, whereas the visible reflectivity band occurring near the 700 nm wavelength spectrally overlaps with nearly horizontal segments of some high-photonic bands characterized by low group velocity.

Accordingly, resonant backscattering should be associated with coupling losses between incident plane waves and slow-light modes inside the PhC^107^. Exploitation of high-photonic band dispersion allows realization of PhC-based structural color materials without the need to downscale their lattice period. Slow-light regions are promising for sensors owing to their increased light–matter interaction.

### Ultimate feature size vs. resolution

The latest advances in nonlinear polymerization-based laser structuring reveal that it is not the exposure conditions but the reacting material itself that is a real limiting factor for the ultimate resolution^108^. First, one should clearly distinguish the difference between the feature size and the resolution—in other words, dimensions of a single feature and resolved separate features^109^. Although nonlinear light-matter interaction-induced modification volume can be squeezed down to tens of nanometers by reducing the applied laser intensity (the exposure dose), it is not very helpful for ensuring scale-down of the resolution^110, 111^. The fabricated structure is revealed by using wet development; the tiny volumes of cross-linked material tend to shrink and become even smaller. Hence, this minimizes the polymerized feature size but hardly improves the resolution.

**Figure 7:**
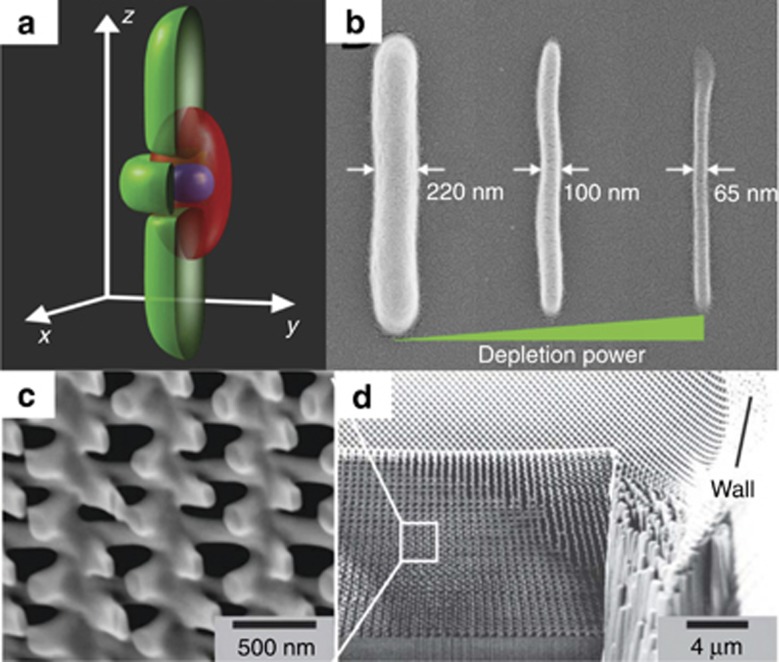
(**a**) A typical DLW STED exposure profile: red volume represents standard focusing exciting the material; green represents a phase mask-generated depletion (bottle beam); violet is the resulting modified volume (excitation is minimized by cutting the side and top-bottom lobes with a shaped depletion beam)^73^. (**b**) Using the same excitation conditions but increasing the depletion power, the feature size shrinks (from left to right, it is minimized from 220 nm down to 65 nm)^73^. (**c**, **d**) Magnified and full-scale image of 3D chiral polarizer structure fabricated employing simultaneous combination of dip-in^50^ and STED techniques^111^. Reproduced with permission from Refs. 73, 111. OSA. All rights reserved.

The stimulated emission depletion (STED) technique, well established in multi-photon microscopy^112^, can be implemented to improve both the features size and the resolution, as shown in Figure 7a and 7b. STED-inspired lithography relies on simultaneous photo-induced activation and deactivation of the polymerization process.

The ultrafast excitation pulse drives the photoinitiator molecules to a long-lived intermediate state that initiates polymerization on a relatively slow timescale. A second laser is employed to quench this intermediate to the ground state, preventing polymerization. By spatial beam shaping, the photomodification can be confined to a well-sub-diffraction-limited small region. This research vector was extensively studied by several research groups; M. Wegener’s and T. Klar’s are among the most active. Their results proved that the feature size can be more confined by optimizing the material for that purpose and applying a dual exposure: an ultrafast excitation and inhibition^113^. Experimentally realized arrangements including phase masks for specific beam shaping are already described in detail elsewhere^111, 114^. Most importantly, it was shown that this approach empowers dramatic improvement of not only the lateral but also the axial resolution^115^. This progress makes the DLW lithography technique even more attractive and versatile for the manufacturing of state-of-the-art photonic as well as biological components^116^. Despite these achievements, the topic of achievable resolution and feature size remains actively debated with the main arguments focused on the mechanisms of polymerization and its kinetics^117, 118, 119, 120^.

## Volumetric Structuring of Dielectrics

The unique 3D capability of fs-laser writing is overviewed next for the creation of new materials and strategic fields of telecommunications^121, 122^ and various biomedical applications^22, 23, 71, 72^.

### Creation of new materials via extreme pressure and temperature conditions

With tight focusing and concentration of light into a small submicron focal spot and by using ultrashort laser pulses *τ*_p_≃100 fs, the intensity reaches several terawatts per square centimeter for small-energy *E*_p_≃100 nJ pulses. For such pulses, self-focusing can be avoided because the pulse power *E*_p_/*τ*_p_≤1 MW/pulse is below the self-focusing threshold inside the dielectric and semiconductor materials; however, the intensity can already surpass the dielectric breakdown threshold.

In multiple ionized plasma and under strongly nonlinear dynamics of electrons and ions in abrupt pressure and temperature gradients, separation of the ions of different masses occurs^123, 124^. After the microexplosion, strong thermal quenching facilitates the recovery of metastable phases of materials. Some of those phases are high-pressure and temperature nanomaterials previously only predicted by modeling. The creation of molecular oxygen in glasses^125^; change of the ionic makeover of complex compounds such as one of the Earth’s most abundant minerals, olivine^126^; fixation of highly unstable amorphous phase of sapphire^127^; and formation and recovery of body-centered cubic-Al inside sapphire, which requires pressures such as in the Earth’s center^128^, are recent illustrative examples.

When a light pulse is squeezed to tens of fs and focused into volumes with cross-sections of sub-wavelength dimensions (hundreds of nanometers) and absorbed at an even smaller volume where dielectric breakdown is created, the energy density reaches levels encountered in the strongest military macro-explosions. A phenomenal scale span over which a hydrodynamical stage of the explosion can be modeled is shown at energies 10^21^ times lower, and time scales are reduced by a factor of 10^7^, creating the same Energy/Volume density^129^. At such exotic *p*, *T* conditions, defects inside glasses and crystals can be easily created at large densities^5, 130, 131, 132^. Such strongly altered regions have different chemical etchability in acidic^133, 134^ and basic^135^ solutions now widely used for micro-fluidics, structural analysis of the micro-/nanostructure of the interaction volume and fabrication of 3D micro-parts in silica and sapphire.

Temporal and spatial chirp control allows additional tunability of light–matter interaction of focused ultrashort laser pulses^136, 137, 138, 139^. Interestingly, a new metal welding technology based on a spark discharge uses an fs-laser-guide for pointing and chirp control of ionization. This is an example of an industrial process in which subtle parameters of fs-laser pulses are at work. Welding of previously incompatible materials, glasses, ceramics and metals with very different thermal expansion coefficients becomes possible using ultrafast lasers^140, 141^.

The generation of a white light continuum using fs-laser pulses in water has proved to result in size homogenization of colloidal nanoparticles made by ablation in water^142^. For example, such colloidal suspensions of Au nanoparticles are stable in time against precipitation without surfactant additives and finds increasing popularity in biomedical sensing because of the pure Au surface accessible for functionalization^143^.

### Passive and active waveguides

Waveguiding regions can be made by direct laser writing inside transparent materials. The guiding occurs in the augmented refractive index locations *n*=*n*_0_+|Δ*n*|, which can be created by stress-induced modifications, defects or densification; *n*_0_ is an unperturbed refractive index of the medium. A fast thermal quenching important for the waveguide recording can be exploited in fs-laser writing. Glass structures typical for the elevated temperatures can be frozen along the written track. A so-called fictive temperature of glass corresponds to the particular glass morphology. In some glasses such as silica, the anomalous behavior is observed when denser phase corresponds to the higher temperature. For silica, the largest refractive index (a mass density) is at *T*_dense_=1500 °C^125^. If waveguide writing conditions are chosen such that the recorded track has a fictive temperature close to *T*_dense_, a waveguide can be formed by fast quenching. Moreover, the waveguiding region does not exert compressive stress to surrounding regions as in most glasses and crystalline materials where the recorded track is expanded and is not waveguiding. The lowest loss waveguiding has been demonstrated in a tempered Gorilla glass^15^. In crystals, formation of strongly localized stress allows birefringence to be engineered^144^ into patterns that can efficiently guide light^145^.

Waveguides written by fs-laser processing performs interferometry and quantum computing and is integrated into microfluidic chips^7^. Chip-integrated optical lanterns fabricated by fs-DLW are demonstrated for transfer of the focal plane image of large optical instruments such as telescopes onto CCD or spectrometer slits with preservation of phase information^146, 147^ with spectral filtering capability via laser-written Bragg gratings^148, 149^. This is achieved via a calculation of the 3D trajectory of the waveguide, which maintains the same length and does not approach other waveguides to avoid crosstalk. To maintain coherence from the image plane to the detector array, single-mode waveguides are fabricated. This newly emerging field is branded as astrophotonics^150^. Active laser-written waveguides have been demonstrated at exotic wavelengths and in a small-footprint glass pieces^151^ for future on-chip and lab-in-fiber integration^152^.

**Figure 8:**
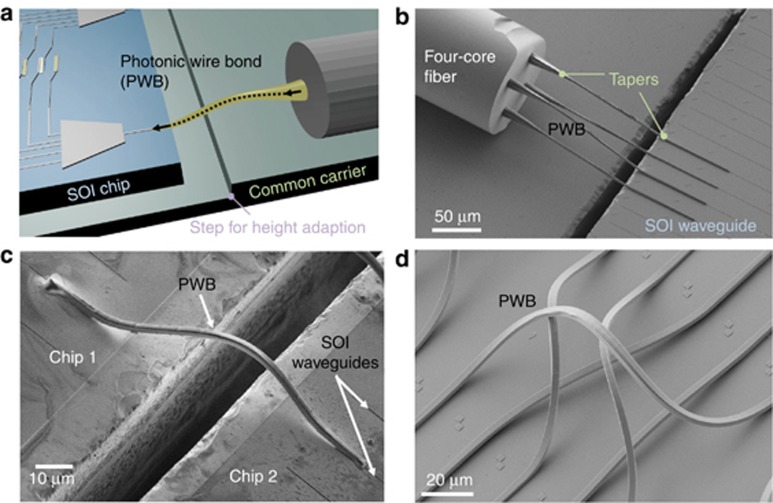
PWB by the 3D DLW lithography approach^121, 122^. (**a**) A principal scheme of the PWB enabling connection of an SOI chip with an optical fiber. (**b**) A four-core optical fiber coupled with SOI waveguides by tapered photonic wires. (**c**) An integrated chip with two SOI waveguides coupled together via freeform PWB; note a spatial (lateral) displacement of the SOI waveguides on the separate chips. (**d**) An example of 3D PWB capability enabling virtually direct signal transferring through different communication platforms. Images courtesy of Prof. C. Koos. (2015) IEEE. Reprinted, with permission, from Ref. 122.

One of the most recent and promising practical advances of 3D-laser writing is emerging in the field of photonic wire bonding (PWB)^122^ illustrated in Figure 8. This approach solves issues regarding the connection of silicon photonic circuits made on semiconductor-on-insulator (SOI) industrial platforms to multicore fibers or lasers by DLW 3D lithography and offers novel concept solutions applicable to chip-scale interconnection. Altogether, this induces a dramatic increase of possibilities for the continuously growing telecommunication industry^153^. Here, 3D-laser-written waveguiding structures can be seen to be serving as wires in former microelectronic circuitry, a very logical step when circuits become photonic instead of electrical.

## Towards Printing Bio-Parts and Smart Implants

The 3D printing of bio-parts^154^, food and even houses is a fiction quickly becoming a reality. The principles of stereolithography (3D fabrication) with light scanned over an absorbing surface of resin that is refreshed constantly as the workpiece is moved in the liquid are now taken forward and cross-pollinated with ideas of jet printing and silkography mastered in the solar cell industry. For the mass production of items larger than 1 mm, those new technologies are presenting what could be considered a disruptive technology^155^; that is, they are very simple with a setup acquisition and maintenance cost up to two orders of magnitude smaller than fs-laser-based tools. Indeed, simple light sources or thermally controlled extrusion through a nozzle with a diameter of tens of micrometers is already delivering fascinating 3D fabrication of biodegradable tissue scaffolds and can prototype larger workpieces^156^—for example, benchtop optic holders. In Figure 2, this would correspond to 100 μm resolution and high productivity ∼10^12^ μm^3^ h^–1^ near the line corresponding to the 3D scaling by Tennant’s law.

**Figure 9:**
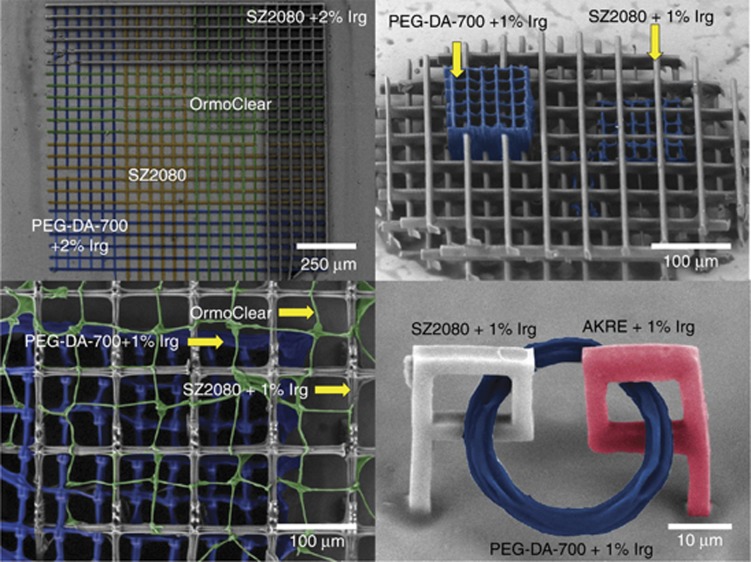
Hybridization of scaffolds made out of different negative-tone photoresists (color-coded for clarity): AKRE—red, SZ2080—gray, PEG-DA-700—blue, Ormoclear—green. Reproduced with permission from Ref. 18. JLPS. All rights reserved.

Figure 9 illustrates the versatility of DLW and the compatibility of different materials that can be combined, exposed and developed for the final 3D complex scaffolds. All materials are transparent with slightly different refractive indexes, helping with the recognition of the earlier fabricated regions in such multiplexed patterns. The control of surface chemistry, wettability and mechanical properties on the mesoscale (from tens of nanometers to tens of micrometers) is currently an active field of research for bio-scaffolds^154, 157, 158^. DLW control of the volume fraction of a polymer in a 3D structure not only changes its permeability but also provides a method to deliver matching mechanical strength of implant and tissue via fracture toughness engineering^159^.

Despite strong competition in 3D fabrication among the different approaches discussed above, a high-precision laser structure will have unique advantages for high-precision scaffold formation where different materials must be combined (Figure 9) or high-resolution feature size patterns^160, 161^ are required. Functioning of the cell membrane receptors for opening drug delivery pathways occurs via 3D morphological changes on the membrane on the scale of tens of nanometers^162^. Currently, this is the resolution achievable by DLW lithography in 3D^40^ and can be combined with standard nanotechnology methods. Bio-related research for understanding fundamental mechanisms on the sub-cellular level will require the highest resolution of laser writing.

**Figure 10:**
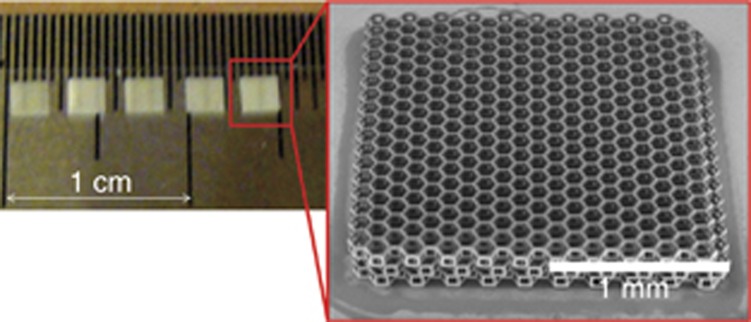
Free (not attached to substrate) macro-3D scaffolds out of SZ2080. Fabrication time 2.5 h/piece with 515 nm/300 fs direct write at 7 mm s^–1^ sample translation synchronized with beam scanning^154^.

**Figure 11:**
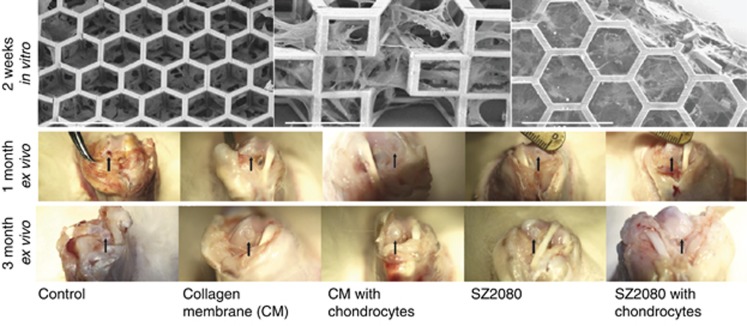
First *in vivo* tests of laser-fabricated scaffold^154^. SEM images of a segment of centimeter-scale membrane tested *in vitro* by pre-growing isolated allogeneic rabbit chondrocytes for 2 weeks; scale bar 100 μm. *Ex vivo* photo images after implantation of laser-fabricated 3D scaffold pre-incubated with chondrocytes and implanted into rabbit’s knee (marked by arrow) after 1 and 3 months; 54 bilateral osteochondral defects 3 mm in diameter were created using a surgical drill at the weight-bearing areas. Reproduced with permission from Ref. 154. IOP. All rights reserved.

On a larger than cellular scale, the biomedical field is actively researching facile methods for fabrication of macro-3D scaffolds (Figure 10). Recently, a preclinical study of 3D artificial micro-structured scaffolds out of hybrid material SZ2080 fabricated using the DLW technique was reported^154^. The created centimeter-scale membrane constructs were tested both *in vitro* by pre-growing isolated allogeneic rabbit chondrocytes and *in vivo* by implantation into rabbit organisms for up to 6 months (followed by *ex vivo* characterization) (Figure 11). Using a surgical drill, 54 bilateral osteochondral defects with a 3 mm diameter were created. Weight-bearing areas of medial femoral condyles were chosen for a preclinical study. *Ex vivo* histological examination shows that specific 3D pore geometry and pre-growth of chondrocytes before implantation significantly improved the performance of the manufactured scaffolds. The achieved biocompatibility was comparable to collagen membranes widely used in surgery. A successful outcome of the study supports the idea to implement DLW into clinical practice for fabricating patient-specific 3D micro-structured scaffolds that, in combination with cell seeding, may be a significant advance in cartilage tissue regeneration. In addition, it can serve as a tool for generating prototype scaffolds of bio-inspired microarchitectures, and their characterization of mechanical properties required for both hard (bone and cartilage) and soft (muscle, skin, etc.) tissue engineering^163^. Recent studies show that by employing the fs-DLW technique, biocompatible and biodegradable materials such as silk can be 3D structured^164^.

Printing via LIFT has already been advanced as a technological solution for the fabrication of microelectronic circuitry^165^, and is applicable for the transfer of biomaterials with low damage when ultrashort laser pulses are used. The printing tape principle is applied to form light-absorbing film with functional materials facing the substrate onto which it will be transferred. When ultrashort laser pulses are used, the transfer gaps can be larger, and more directional LIFT is performed owing to the efficient and spatially well-controlled energy deposition^166^.

Apart from 3D printing by laser-assisted processing and LIFT, ultrashort laser applications in surgery is another vast field with huge potential. Very precise energy delivery in eye surgery^167^ and focusing through a turbid medium using spatiotemporal focusing^168^ are just a few examples in which unique control over the delivery of ultrashort laser pulses to the modification/surgery point can be exercised.

Direct write additive manufacturing processes are unique in flexibility yet inherit the underlying limitation of being time consuming owing to the serial nature of point-by-point structuring^169^. The current development of fs-pulsed industrial high-repetition-rate lasers limits the DLW technique by either an efficient sample positioning or beam scanning for high-throughput manufacturing. Recently, a continuous generation of monolithic polymeric parts was demonstrated using stereolithography-based 3D printing, which is believed to allow print manufacturing of parts in minutes instead of hours^170^. The control of the polymerization mechanism using an oxygen-permeable membrane is responsible for faster 3D printing by up to two orders of magnitude.

## Complex Light Fields

Experimental studies have shown that complex 3D microstructures can be polymerized by multiple foci created and translated by a holographic spatial light modulator (SLM)^171^ and can be exploited up to the diffraction-limited spatial resolution^172^. This enables efficient generation of up to 10 independent foci for parallel holographic fs DLW. Currently, a common SLM ensures a 60 Hz refresh rate, but it can be further increased by the application of faster modulators that are commercially available (200 Hz) with even higher rates expected in the near future^173^.

When SLM is too slow for an application, an optical element can be made for a specific phase pattern required for laser fabrication. Recently, for material processing using fs Airy beams, such an optical element was made in glass by a form-birefringent pattern^174^. Airy beams are advantageous for glass scribing, cutting and complex edge and corner formation on a workpiece. With the Airy beam scribing and glass-breaking, a spontaneous self-detachment of a fiber-like structure was induced, offering an alternative way to fabricate glass cantilevers^174^.

Development of beam shaping is leading to solutions in which simultaneous control of timing, positioning, phase and polarization of the one or several laser pulses is performed. Surface or bulk modification of transparent materials becomes less distinct in the case of high-intensity ultrashort laser pulses. High intensity can be created inside the material at its optical transparency window as well as in subsurface regions. By creating a high-density electronic excitation, a newly introduced interface then acts as a transient meta-surface that responds to different parameters of the incoming light: polarization, spatial and temporal chirp, ponderomotive action at high intensity and angular momentum of the beam.

### Nanotexturing by polarization control

In many cases, it is already proven that peak intensity is a more important parameter than the total energy dose for the required modification. The influence of other parameters, however, can also be very important—for example, the polarization is actively studied in control of ablation and formation of (nano-)ripples.

The structuring of materials by ultrashort laser pulses demonstrated new morphologies in nanotexturing of the surface and volume by self-organized quasi-periodic structures—ripples^29, 84, 175, 176^. Photo-excitation of electron-hole solid-state as well as breakdown plasmas are leading to the formation of sub-wavelength period ripples via imprint of a surface wave on the substrate–plasma interface. A surface wave on a plasma is created when the real part of dielectric permittivity ℝ*ε*^∗^<−*n*^2^, where *n* is the refractive index of the dielectric medium and a phase matching between the surface wave and the **k**-vector of photons is met. Because the TM-mode with E-field polarized in the plane of incidence (p-polarization) is the most efficient in launching the surface wave, most of the observed ripples on the surface have an orientation perpendicular to the E-field (or a wave vector **k** of the ripple-grating is parallel to **E**). Hence, polarization becomes one of the key control parameters for surface and in-volume formation of ripples, which is shown next for the case of arbitrary and simultaneous control of several beamlets.

### Dynamic real-time beam shaping

**Figure 12:**
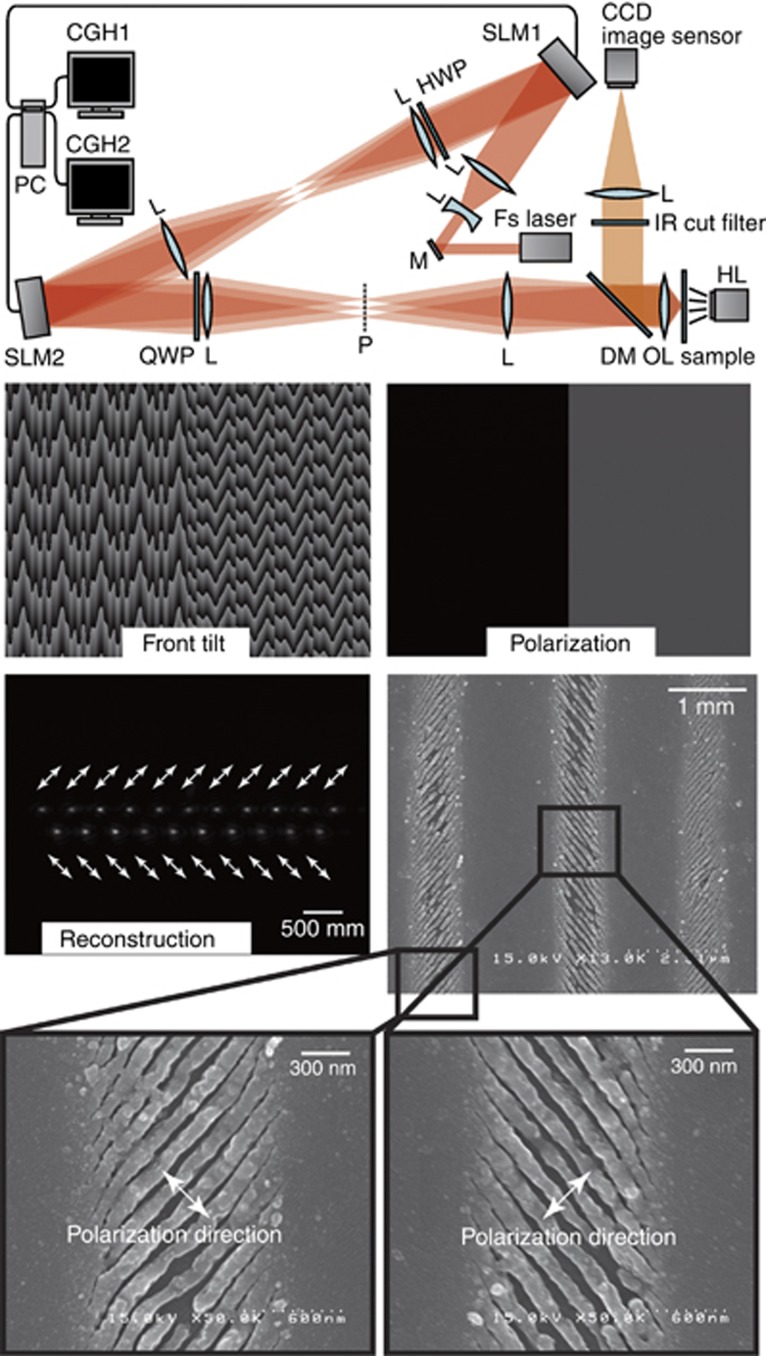
Optical setup with two SLMs for independent control of the wave front tilt and polarization. HWP, QWP and SLM phase masks allows an arbitrary control of the polarization and wave front tilt. SEM images of the single-pass scan ablation of indium tin oxide with adjacent lines polarized differently. Reproduced with permission from Ref. 169. Taylor & Francis Ltd, http://www.tandfonline.com. All rights reserved.

The polarization and wave front tilt control provides a way to manipulate surface nanotexturing and ripple formation. Figure 12 shows a method to prepare arbitrary polarization, including vectorial beams using SLMs and polarizing optics: *λ*/4 and *λ*/2 quarter- and half-waveplates QWP and HWP, respectively^169^. The polarization of the output beam **E**_out_ is given:


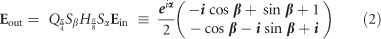


where **E**_in_ is the Jones vector of the linearly polarized input beam, the subscripts *H* and *Q* are the azimuthal angles of the respective HWP and QWP plates, and *α*, *β* are the phase retardation applied to the pair of SLMs, respectively. The wave front and polarization of the output beam are independently controlled by choosing *α* and *β*. For 3D fabrication, SLMs^74, 177^ are also useful for compensation of aberrations—in particular, spherical—due to a refractive index mismatch between the substrate and the workpiece and a different depth of focus^178^. Airy beams^179^ can be made using SLMs and are very promising in laser cutting applications similarly to the Bessel–Gauss beams^180^.

Different incarnations of polarization and phase control setups using SLMs, diffractive optical elements and axicon lenses can be used to generate light intensity distributions, such as doughnut, Gaussian–Bessel^181, 182^ or flat-top. Experimenting with such beams is a currently active field of investigation along with the control of spatial-temporal focusing^168^. Arrays of beams with individual control of polarization, intensity profile and front tilt can speed up fabrication and more efficiently use laser power in industrial applications^183^.

## Conclusions and Outlook

This mini-review of the state of the art and emerging applications shows the maturity of the ∼20-year-old field. The productivity of fs-laser fabrication required for practical applications is demonstrated to be consistent with technologically matured fabrication methods used in 2D planar microelectronics and is now challenging true 3D microfabrication. A comparison with the production of basic 3D building blocks in a living cell—proteins—that acquire 3D conformation encoded in a linear RNA or DNA sequence is shown to follow similar scaling to the fastest examples of direct laser writing used for fabrication of fully functional 3D patterns: optical gratings, bio-scaffolds and PhCs. The 3D wiring of future telecommunication circuitry by 3D waveguides is another successful emerging application.

Biomedical applications involving phenomena from the receptor scale of ∼10 nm up to implantable 3D scaffolds with cross-sections *>*1 cm require adequate biocompatible materials and processing approaches to structure them. Here, we show that modern laser processing with ultrashort laser pulses can address this challenge from the resolution, productivity and materials points of view. By controlled laser curing, photoinitiators become obsolete^56^, and light–matter interaction is controlled solely by parameters of light. This feature opens the window to the use of tested biocompatible materials without bio-toxic additives and polymerization promoters^28^. In the future, proteins can be used for optical applications and laser structured from micro- to macroscale patterns or devices^184^.

Advances in the understanding of light–matter interaction peculiarities in different materials at diverse conditions of ultrashort pulse delivery onto the surface or inside transparent materials are helping simplify material processing protocols. The reliability of lasers and maturity of fs-pulse control in terms of duration, spatiotemporal chirp and focusing, and polarization meets the 24/7 requirements of industrial applications. Time will tell the extent to which these methods will become niche for this technology in real-world applications within the next few years. Rival technologies based on long pulses and continuous laser radiation along with fast and precise workpiece positioning and 3D printing are ‘disruptive technologies’^155^. Innovative disruptive technologies have proved in the past to be commercially successful. Could the demonstrated fabrication at the feature size of ∼100 nm and submicron spatial resolution (a separation between individual features) delivered at the millimeter scale be a ‘tipping point’^185^ for industrial adaptation of fs-laser fabrication? The millimeter-size 3D scaffolds can be made out of the same material and laser cured without toxic initiators for implant applications within a practical time frame. This span of mesoscales from the formation of nano-features to implantable structures is a unique and appealing characteristic of 3D printing by DLW. The Tennant scaling law shows that this 3D technology is mature in terms of its industrial productivity.
